# Percutaneous intervention versus surgery in the treatment of common femoral artery lesions: study protocol for the prospective, multi-center, randomized PESTO-CFA trial

**DOI:** 10.1186/s13063-024-08219-1

**Published:** 2024-06-08

**Authors:** Aljoscha Rastan, Tanja Böhme, Thomas Zeller

**Affiliations:** 1 Department of Angiology, Cantonal Hospital Lucerne, Lucerne, Switzerland; 2 Department of Cardiology and Angiology, Medical Center, University of Freiburg, Freiburg, Germany

**Keywords:** Common femoral artery, Surgery, Atherectomy, Drug-coated balloon, Angioplasty, Peripheral artery disease, Randomized controlled trial

## Abstract

**Background:**

Endovascular therapy has become established as a first-line therapy in most arterial regions. However, open vascular surgery (endarterectomy) remains the treatment of choice for common femoral artery (CFA) lesions. The aim of this study is to investigate the acute and mid-term results of directional atherectomy plus drug-coated balloon (DCB) in comparison to endarterectomy in treatment of de novo arteriosclerotic CFA lesions.

**Methods:**

This prospective, randomized, multicenter non-inferiority study will enroll 306 participants with symptomatic (Rutherford category 1 to 5) de novo stenosis of the CFA including the bifurcation. Patients eligible for both treatment groups could be included in this 1:1 randomized trial. Primary efficacy endpoint is patency of the target lesion at 12 months defined as restenosis < 50% without the need of clinically driven target lesion revascularization (cdTLR). Primary safety endpoint is a combined endpoint including death, myocardial infarction, major or minor amputation of the target limb, and peri-procedural complications at 30 days. Secondary endpoints include primary patency of the target lesion at 6 and 24 months, secondary patency, cdTLR 6, 12, and 24 months, change in ankle-brachial index, and Rutherford-Becker class at 6, 12, and 24 months. Limb salvage, change in quality of life measured by Walking Impairment Questionnaire, and major adverse events including death, myocardial infarction, and minor or major amputation of the target limb will be determined at 6, 12, 24, and 36 months.

**Discussion:**

Endovascular treatment of CFA lesions is still a matter of debate. Few studies compared modern endovascular therapy methods against the so-called gold standard surgical endarterectomy so far. Based on recent positive results, this study aims to confirm non-inferiority of a “leaving nothing behind” endovascular approach combining directional atherectomy and DCB compared to surgical therapy.

**Trial registration:**

ClinicalTrials.gov NCT02517827.

## Administrative information

Note: the numbers in curly brackets in this protocol refer to SPIRIT checklist item numbers. The order of the items has been modified to group similar items (see http://www.equator-network.org/reporting-guidelines/spirit-2013-statement-defining-standard-protocol-items-for-clinical-trials/).

**Table:** 

Title {1}	Percutaneous Intervention versus Surgery in the Treatment of Common Femoral Artery Lesions: Study protocol for the prospective, multi-center, randomized PESTO-CFA trial
Trial registration {2a and 2b}	www.ClinicalTrials.gov NCT02517827
Protocol version {3]	V5.3, 12 May 2023
Funding {4}	The study receives unrestricted funding by the manufacturer of the atherectomy device and drug-coated balloon (Medtronic, Mansfield, USA).
Author details {5a}	Prof. Dr. Aljoscha Rastan, Department of Angiology, Cantonal Hospital Lucerne, Lucerne, Switzerland Dr. Tanja Böhme, Department of Cardiology and Angiology, Medical Center – University of Freiburg, Germany Prof. Dr. Thomas Zeller, Department of Cardiology and Angiology, Medical Center – University of Freiburg, Germany
Name and contact information for the trial sponsor {5b}	Universitäts-Herzzentrum Freiburg-Bad Krozingen, Germany Sponsor Representative: Prof. Dr. Thomas Zeller, Universitäts-Herzzentrum Freiburg-Bad Krozingen, Angiologie Campus Bad Krozingen, Südring 15, 79189 Bad Krozingen, Phone: + 49 7633 4020, thomas.zeller@uniklinik-freiburg.de
Role of sponsor {5c}	Final authority for study design, collection, management, analysis and interpretation of data, writing of the report, and decision to submit the report for publication rests with the study sponsor (Universitäts-Herzzentrum Freiburg-Bad Krozingen).

## Introduction

### Background and rationale {6a}

Percutaneous transluminal angioplasty (PTA) has been shown to be a successful method of treating peripheral artery disease (PAD) and is established as one of the first line therapy options in most arterial regions [1].

Today, the use of stents, directional atherectomy, and drug-coated balloon angioplasty (DCB) leads to favorable mid-term results after endovascular procedures in the femoropopliteal arteries [1–3]. However, concerning the common femoral artery (CFA) lesions, open vascular surgery (endarterectomy) is still considered the treatment of choice. The reported 1-year primary patency rates after endarterectomy of the CFA range from 85 to 95% [4]. However, peri-procedural major adverse events in up to 5% and minor complications in up to 20% were documented after open surgery, including infection, acute re-occlusion, hematoma, and nerve damage [4–6].

Today, endovascular therapy of the CFA including the bifurcation is mainly performed in exceptional cases like in patients with multi-level disease, patients unfit for surgery, or patients who refuse an open surgical procedure.

The TECCO trial is the only prospective randomized controlled trial (RCT) to date comparing endovascular stent angioplasty with surgery. At 24 months, sustained clinical improvement, primary patency, target lesion, and limb revascularization rates did not differ between the two groups [7].

Nevertheless, there are concerns regarding CFA stent implantation because of the potential mechanical stress exposed in a motion segment.

Combining (directional) atherectomy and balloon angioplasty with or without drug coating is an endovascular option for following the “leaving nothing behind” concept. However, there is only limited data available concerning the performance of atherectomy in CFA disease [8, 9].

Whether open surgery is superior to these current endovascular strategies was not yet investigated in a prospective RCT.

The aim of this multicenter RCT is to investigate the acute and mid-term results of directional atherectomy followed by DCB angioplasty with provisional stenting in comparison to open surgery (endarterectomy) in treatment of de novo arteriosclerotic CFA lesions including the femoral bifurcation. Noninferiority of the endovascular procedure in comparison to the open surgery approach is assumed.

### Objectives {7}

The objective of this study is to compare the performance of directional atherectomy combined with DCB angioplasty over vascular surgery in CFA lesions in a prospective, multi-center, randomized clinical trial. The primary objective of this trial is to assess whether primary patency at 1 year after endovascular treatment is comparable to that after open surgery. Primary patency is defined as freedom from restenosis (< 50% lumen diameter, measured by duplex-ultrasound, peak velocity ratio (PVR) cut off value < 2.4 [10] or angiographic stenosis ≤ 50% of CFA lumen diameter), without the need of clinically driven target lesion revascularization (cdTLR).

Primary safety objective is to assess the freedom from a combined endpoint (including death, myocardial infarction, major or minor amputation of the target limb, and peri-procedural complications) at 30 days after either endovascular treatment or open surgery.

Secondary objectives are to compare secondary patency rate, cdTLR rate, limb salvage rate, MAE rate, change in quality of life measured by Walking Impairment Questionnaire, and change in ABI and Rutherford-Becker class.

### Trial design {8}

The PESTO-CFA study is a prospective, randomized, multicenter study comparing atherectomy with drug-coated balloon angioplasty (with provisional stent placement) versus open surgery in the treatment of CFA lesions including the femoral bifurcation to evaluate the technical and clinical results for 36 months follow-up. The study hypothesis is the “non-inferiority” of endovascular therapy to surgery with regard to the primary endpoint.

The Albert-Ludwigs University ethics committee, Freiburg, Germany, approved the study (Nr. 399/15 (MPG §23b, dated 12 May 2016). The study is registered with ClinicalTrials.gov (NCT02517827).

## Methods: participants, interventions, and outcomes

### Study setting {9}

The PESTO-CFA study will be realized at different clinical sites with endovascular and surgical departments in Germany and Switzerland (the sites are listed in the Appendix). Depending on the treatment standards of the respective clinic, treatment can take place on an inpatient or outpatient basis.

### Eligibility criteria {10}

Subjects must satisfy all of the inclusion criteria and none of the exclusion criteria in order to be enrolled in the study.

Inclusion criteria.

- 1. Age ≥ 21 years.
- 2. Signed informed consent.
- 3. Patients with PAD (diagnosed with duplex ultrasound and/or CT angiography) Rutherford-Becker class 1–5 [11]
- 4. De novo occlusion or stenosis of the CFA or occlusion of the CFA-bifurcation [including up to 10 mm of the origin of the deep femoral artery (DFA) and/or the superficial femoral artery (SFA) (diagnosed with duplex ultrasound and/or CT angiography).
- 5. Patent distal portion of the popliteal artery and at least *one* patent infrapopliteal artery to the foot.
- 6. No inflow stenosis (> 50%) of the iliac arteries. Treatment of the ipsilateral common iliac artery and *proximal part* of the external iliac artery is allowed to be treated during index procedure.
- 7. The target lesion can be treated by surgery and by endovascular therapy (interdisciplinary colloquium consensus).

In addition, in case of endovascular therapy:

- 8. Guidewire has to cross the target lesion intraluminally, without the use of a re-entry device.

### Exclusion criteria

1. CFA lesions that extend more than 10 mm into the DFA and/or the SFA
2. Previous surgery or endovascular therapy of the CFA
3. Thrombotic stenosis or occlusion of the CFA
4. Aneurysm of the ipsilateral common iliac-, external iliac artery, or the target lesion
5. Stenosis > 50% or occlusion of distal part of the ipsilateral external iliac artery
6. Participation in another study
7. Coagulopathy
8. Pregnancy
9. Contraindication to antiplatelet therapy or heparin
10. Factors which might influence the follow-up measurements (e.g., immobilization, compliance)
11. Life expectancy < 24 months
12. Patients on dialysis
13. Known contrast agent allergy
14. Stroke or myocardial infarction < 30 days prior to scheduled index procedure
15. Thrombolysis up to 72 h prior to scheduled index procedure
16. Contraindications concerning the use of the SilverHawk/TurboHawk/HawkOne atherectomy device or the Admiral-/PacificINPACT balloons as described in the instruction for use (IFU)

### Who will take informed consent? {26a}

Only patients suitable for both treatment groups could be included in this trial. A sub-investigator approved for the study will obtain informed consent. The informed consent form must be signed by the subject or the subject’s legally authorized representative before participation in the study. Documentation of the date informed consent was obtained, and a notation that a signed copy was given to the subject should be recorded in the subject’s records.

Signed consent forms must remain in each subject’s study file and must be available for verification by study monitors at any time. Documentation of the date informed consent was obtained, and a notation that a signed copy was given to the subject should be recorded in the subject’s patient records.

### Additional consent provisions for collection and use of participant data and biological specimens {26b}

There are no additional consent requirements for the collection and use of participant data and biological samples. Biological samples will not be analyzed. Consent for data collection is given by agreeing to participate in the study.

## Interventions

### Explanation for the choice of comparators {6b}

Surgical reconstruction of CFA lesions is considered the standard of care. Therefore, an endovascular technique similar to the surgical technique removing obstructive material was chosen with directional atherectomy being considered the most efficient atherectomy device. Atherectomy is used to reduce the risk of re-coil and/or dissection of the CFA and thus avoid additional stent implantation. A. “leave no metal behind” strategy is purposed, as this preserves the native CFA as an arterial access for percutaneous interventions and does not complicate open surgical treatment of this vascular segment that may be indicated in future.

Based on the favorable outcome in superficial femoral disease treatment, DCB was considered as the best adjunct to atherectomy for achieving optimal acute and long-term treatment results with a limited need for implanting a permanent scaffold what is allowed only for bail-out situations. Only patients/lesions suitable for both treatment groups can be included in this trial. Patients will be randomized 1:1 to endovascular or surgical therapy.

### Intervention description {11a}

Directional atherectomy *prior* to DCB angioplasty of the target lesion is mandatory. In this study, only the Turbo-/SilverHawk or HawkOne directional atherectomy device (Medtronic/Covidien, Mansfield, USA) will be used for treatment of the target lesion. The selection of the device model will be at the discretion of the treating physician and atherectomy procedure will follow the steps described in the Instructions for Use (IFU) enclosed with each catheter. The atherectomy *must* be performed with protection of the outflow vessel (SFA or DFA). The placement of the protection device into the SFA (if patent) is recommended. Residual stenosis following atherectomy must be documented according to the angiographic core laboratory protocol. Furthermore, additional treatment of the SFA and/or DFA (beyond 1 cm of the origin, see inclusion criteria) is at the discretion of the treating physician.

The study utilized IN.PACT Pacific and IN.PACT Admiral balloons (Medtronic GmbH, Meerbusch, Germany) DCBs. These DCBs are paclitaxel-eluting balloon catheters for PTA procedures. It is a single use, sterile, and minimally invasive balloon catheter to dilate vascular lesions. After an inflation of 3 min, the balloon is deflated and retrieved from the body. The IN.PACT Admiral DCB has a usable catheter length of 80 cm and 130 cm and is compatible with a 0.035″ guidewire. The IN.PACT Admiral DCB is available in lengths of 40, 60, 80, 120, 200, and 250 mm. The IN.PACT Pacific DCB has a usable catheter length of 90, 130, and 180 cm lengths and is compatible with a 0.018″ guidewire. It is available in the lengths 40, 60, 80, and 120 mm. These DCBs are intended for use as a PTA balloon catheter to dilate vascular lesions, for the purpose of improving limb perfusion and decreasing the incidence of restenosis.

The selection of the diameter and the length of the DCB for target lesion treatment will be at the discretion of the physician. Covering the complete CFA lesion (including the segments treated with atherectomy) with DCB is mandatory.

If post-DCB residual stenosis, as measured by angiography, is ≤ 30%, then procedure success as determined by the investigator has been achieved. If a residual stenosis of > 50% remains, bail-out stent placement is recommended; the stent choice is left to the discretion of the operator.

An angiogram of the treated segment must be recorded for subsequent core laboratory analysis of the post-procedure residual stenosis.

The end of the procedure is defined as the time after a complete angiogram, including runoff, has been performed and the last guidewire and catheter have been removed. The kind of access site closure is left to the treating physician’s discretion.

Concomitant medication use and any serious adverse events that may occur during the procedure must be documented.

The open endarterectomy technique incorporates a longitudinal arteriotomy that traverses the length of the diseased common femoral artery, through which a standard endarterectomy is performed under direct vision. The artery is subsequently closed with a patch angioplasty technique using saphenous vein (preferential), bovine pericardium (preferential), or alternative patch materials to preserve vessel lumen diameter.

Furthermore, additional treatment of the SFA and/or DFA (beyond 1 cm of the origin, see inclusion criteria) is at the discretion of the treating physician.

### Criteria for discontinuing or modifying allocated interventions {11b}

If residual stenosis is > 30% following adjunctive DCB, the procedure success endpoint has not been reached, and an additional plain old balloon angioplasty (5 min)—preferentially with a bigger sized balloon compared to the DCB—is recommended. In case of persisting treatment failure after additional balloon angioplasty (e.g., residual stenosis > 30%, flow-limiting dissection, re-coil), additional stent placement (bare metal stent only) has to be considered.

In the event of perforation, long-lasting balloon angioplasty (> 5 min) is recommended. Persistent bleeding can be treated with a covered stent (e.g., Viabahn) *after* DCB angioplasty.

The stent or stent-graft should be oversized by at least 1 mm according to the reference vessel diameter of the target vessel. Post-dilatation of the nitinol stent will be at the discretion of the physician.

### Strategies to improve adherence to interventions {11c}

There is no strategy to improve adherence as there is only one study intervention, surgery, or endovascular therapy.

### Relevant concomitant care permitted or prohibited during the trial {11d}

All patients (in both study groups) receive 100 mg acetylsalicylic acid (ASA) daily. If a patient was not taking ASA prior to the study procedures, a 500-mg ASA loading dose will be administered before the intervention. In addition, patients randomized to the endovascular therapy (group 1) will receive a loading dose of clopidogrel (1 × 600 mg p.o.) on the day of the intervention, followed by a daily dose of 75 mg for a minimum duration of at least 4 weeks. The use of clopidogrel in patients randomized to open surgery (group 2) will be at the discretion of the treating physician.

Patients on oral anticoagulation will receive an additional antiplatelet therapy with ASA 100 mg or clopidogrel 75 mg.

### Provisions for post-trial care {30}

After completion of the study examinations, regular 1-year follow-up examinations of the disease are recommended and offered to all study participants.

### Outcomes {12}

The* primary efficacy endpoint* of this study is primary patency of the target lesion at 12 months (restenosis < 50% lumen diameter, measured by duplex-ultrasound, PVR cut off value < 2.4 or angiographic stenosis ≤ 50% of CFA lumen diameter), without the need of cdTLR. Concerning the primary endpoint (target lesion primary patency at 1 year), the “non-inferiority” hypothesis is assumed.

The* primary safety endpoint* is a combined endpoint including death, myocardial infarction, major or minor amputation of the target limb, and peri-procedural complications at 30 days.

### Secondary endpoints

- Primary patency of the target lesion at 6, and 24 months
- Secondary patency of the target lesion at 6, 12, and 24 months
- Limb salvage at 6, 12, 24, and 36 months
- Change in quality of life measured by Walking Impairment Questionnaire (WIQ) 6, 12, 24, and 36 months
- Target lesion revascularization rate at 6, 12, 24, and 36 months
- Change in ABI and Rutherford-Becker class at 6, 12, and 24 months
- Major adverse events including death, myocardial infarction, and minor or major amputation of the target limb at 6, 12, 24, and 36 months
- Cost analysis of index procedure and follow-up procedures due to re-hospitalizations at 24 months

### Participant timeline {13}

**Fig. 1:**
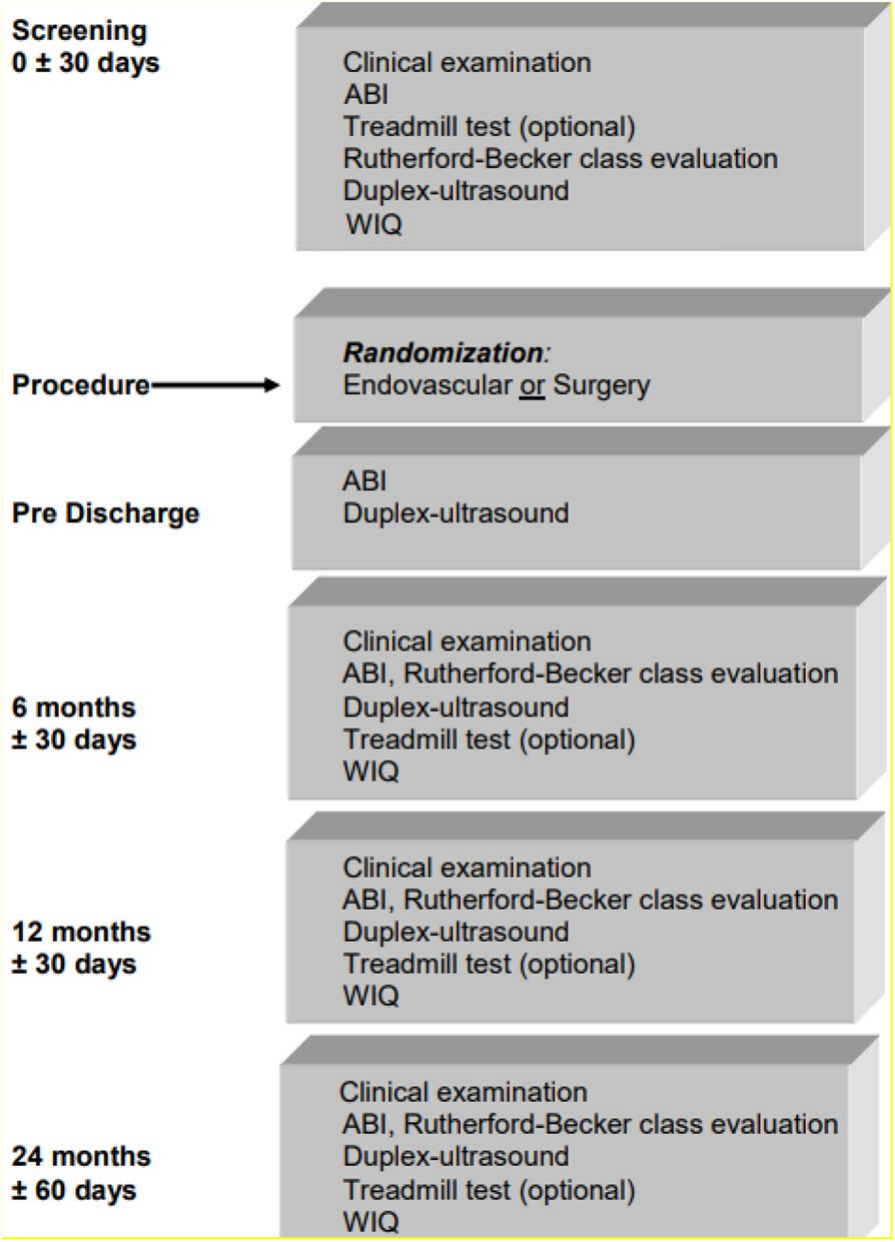
Course of the study. ABI, ankle-brachial index; WIQ, Walking Impairment Questionnaire

After the index procedure, there will be follow-up visits at 6, 12, and 24 months. Follow-up telephone calls will be made at 36 months. The course of the study is shown in Fig. 1.

### Sample size {14}

Based on the available data the primary patency rate at 12 months after successful endovascular therapy with directional atherectomy and DCB is estimated at 80% [2]. Assuming a comparable primary patency of 90% after open surgery [alpha = 0.05 (one-sided) and beta = 0.2, proportion = 0.8, margin =  − 0.2], 260 patients must be included in this trial to reveal the non-inferiority of the treatment groups. Considering a lost-to-follow-up rate of 15%, 306 patients (153 patients in each group) have to be enrolled. Initial estimated enrollment time was 2 years but had to be extended due to the COVID-19 pandemic.

### Recruitment {15}

Patients will be recruited at all centers through the surgical and angiology departments according to local referral practice.

## Assignment of interventions: allocation

### Sequence generation {16a}

For each patient, the investigator receives one sealed envelope containing the assignment to one of the treatment groups. The allocation sequence is determined by drawing lots. The date of opening of the envelope, the treatment group, and the patient initials must be documented. The randomization will be carried out as a block randomization into the two treatment groups, stratified according to the participating center. The participating study centers are blinded in terms of the block size. Only after colloquium consensus [Colloquium consensus: The study investigators (vascular surgeon and the endovascular specialist) review the medical history and examination findings of the patient with CFA disease and agree that the patient is eligible for both treatment modalities] randomization is allowed. Randomization takes place in a 1:1 ratio.

Group 1: Endovascular therapy consists of directional atherectomy and DCB angioplasty with provisional stenting.

Group 2: Open surgery consists of endarterectomy, with or without patch plasty.

### Concealment mechanism {16b}

In order to ensure concealment, sealed envelopes containing the treatment allocation will be provided by the principal investigator.

### Implementation {16c}

For each patient, the investigator receives one sealed envelope containing the assignment to one of the treatment groups. The allocation sequence is determined by drawing lots. The draw is carried out by two persons who are not involved in the study procedures. The date of opening of the envelope, the treatment group, and the patient initials must be documented. The randomization will be carried out as a block randomization into the two treatment groups, stratified according to the participating center.

“After the colloquium consensus and assignment to one of the treatment arms, the investigator of the corresponding discipline is responsible for the planning and implementation of the study procedure.”

## Assignment of interventions: blinding

### Who will be blinded {17a}

Randomization is performed as block randomization into the treatment groups. The participating study centers are blinded with regard to the block size. Due to the differences in revascularization techniques, neither the physician nor the patient will be blinded for the allocated study cohort.

### Procedure for unblinding if needed {17b}

Not applicable. Due to the study design, blinding is not possible.

## Data collection and management

### Plans for assessment and collection of outcomes {18a}

The course of the study is shown in Fig. 1.

- Angiography: An angiogram of the treated segment must be recorded for subsequent core laboratory analysis of the post-procedure residual stenosis
- Duplex ultrasound: Duplex-ultrasound of the target limb arteries should be performed prior to index procedure, pre-discharge, and 6, 12, and 24 months following index procedure. The results must be documented on CD and should include:
- Measurement of the PSV and diameter in a healthy section of the distal portion of the external iliac artery
- Measurement of the PSV within the target lesion. The quotient of PSV within the target lesion and PSV in the external iliac artery gives the PVRprox.
- The quotient of PSV within the target lesion and PSV in the proximal portion of the superficial femoral artery the PVRdist.

PSVR > 2.4 indicates diameter restenosis of > 50% [10] and loss of primary patency. The evaluation of the duplex-ultrasound examinations (including pictures and measurements) will be performed by the independent core laboratory “Black Forest,” Bad Krozingen, Germany.

- Rutherford category: The evaluation of the Rutherford-Becker class based on the walking distance should take place prior to index procedure, pre-discharge, and 6, 12, and 24 months following index procedure
- ABI will be measured: prior to index procedure, pre-discharge, and at 6, 12, and 24 months following index procedure. The ABI measurement should be performed at rest as also after exercise
- Evaluation of the walking distance: The walking distance will be documented using the “Walking Impairment Questionnaire” (WIQ) prior to index procedure, pre-discharge, and 6, 12, 24, and 36 months after index procedure is required
- Treadmill test (optional): A treadmill test should be performed prior to index procedure, pre-discharge, and 6, 12, and 24 months following index
- Medication: A documentation of the antiplatelet therapy, the oral anticoagulation, cilostazol, statins, and angiotensin converting enzyme inhibitors/angiotensin receptor blockers prior to index procedure, pre-discharge, and 6, 12, and 24 months after index procedure is required

### Plans to promote participant retention and complete follow-up {18b}

Before discharge, patients receive all their follow-up visits on an appointment card. Before each follow-up visit, patients are reminded of their appointment by telephone call. Participants will be asked to answer questions about their health status by telephone if they are unable or unwilling to visit the site.

### Data management {19}

All study-specific records as well as the patient files from all the patients who participate in the study are stored and controlled through the investigator site.

Data will be collected on a case report form (CRF). Copies of these documents are included in the application:

1. Subject ID code list
2. Shipping records

The data input is constantly. All data from all participating study sites are entered into a joint database and evaluated centrally by the data progressing agency (StarConsult GmbH, Magdeburg, Germany).

Clinical trial records and documents must be kept for at least 10 years.

### Confidentiality {27}

All information about participants will be kept strictly confidential by the investigators and trial staff. Data will be protected from unauthorized access. Patient data is anonymized and stored on a computer with restricted access and password protection.

### Plans for collection, laboratory evaluation, and storage of biological specimens for genetic or molecular analysis in this trial/future use {33}

This is not part of the study design.

## Statistical methods

### Statistical methods for primary and secondary outcomes {20a}

All analyses are performed using the software SAS 9.4 (SAS Institute Inc., Cary, NY, USA) and are implemented by the procedure PROC POWER.

The collected data are saved on a personal computer (operating system Windows 10).

The treatment groups (endovascular therapy/surgery) are reviewed using standardized statistical procedures. The treatment groups are screened on significant difference regarding the primary and secondary issue.

A significance level of *P* < 0.05 can be considered as significant.

Data of patients who receive at least one of the two treatment methods will be analyzed as randomized (intention-to-treat, ITT set). For all statistical tests, a 2-sided significance level of 5% will be used. The primary endpoint will be analyzed using the per protocol set. For sensitivity analyses, “intention-to-treat” and “treatment received” analyses will be performed.

For the primary endpoint analysis, the proportion of patients with patent target lesion at 12 months using a cut-off value of PVR ≤ 2.4 in duplex-ultrasound is to be determined. A TLR between the procedure (baseline) and 12 months is considered a restenosis. The rate of restenosis in the endovascular therapy group (group 1) will be evaluated using its lower confidence limit (alpha = 0.05, one-sided) in comparison with the non-inferiority margin. This margin is the maximum of 70% and the rate of restenosis in the open surgery group (group 2) minus a delta of 20%. A logistic regression analysis will be performed to identify important covariates for restenosis. Concerning the secondary endpoints, the Fisher’s exact test, chi-square test, or Wilcoxon rank-sum test will be used to compare differences between treatments. Event-free survival after the procedure, time to restenosis, and time to first TLR will be analyzed using Kaplan–Meier estimates.

### Interim analyses {21b}

According to the study protocol, no interim analysis is planned.

### Methods for additional analyses (e.g., subgroup analyses) {20b}

Due to the number of study patients (*n* = 306), no subgroup analyses are planned.

### Methods in analysis to handle protocol non-adherence and any statistical methods to handle missing data {20c}

Time-to-event analyses will be used to involve missing information.

Furthermore, no imputation of missing values is planned. Thus, complete cases will be analyzed per visit and all endpoints will be evaluated on observed data.

### Plans to give access to the full protocol, participant-level data, and statistical code {31c}

The complete protocol, the participant-level data, and the statistical code can be accessed on request.

## Oversight and monitoring

### Composition of the coordinating center and trial steering committee {5d}

A data safety monitoring board (DSMB) comprised of three experts from appropriate disciplines (endovascular specialist, vascular surgeon, and biostatistician) will serve as an advisory panel to the principal investigator.

Of primary concern to the DSMB would be the rate of adverse events, and, therefore, the DSMB will review accumulating safety data to monitor for evidence of trends that would warrant modification or termination of the study.

### Composition of the data monitoring committee, its role and reporting structure {21a}

The CRO (contract research organization) is VascuScience in Leipzig, Germany.

During the study duration, the data quality and the protocol conformity will be reviewed (monitoring) and guaranteed by repeated monitoring visits at all involved study sites.

All individual-related data are subject to the regulations of data protection and rules of professional secrecy. The monitor has the responsibility to stick to the above-mentioned regulations.

1. Monitoring will be performed on a routine basis to assess and report:
  - That the rights and well-being of subjects are being protected
  - That the reported trial data are accurate, complete, and verifiable from source documents
  - That the conduct of the trial is following the currently approved protocol and any approved amendments, with good clinical practice (GCP), with the applicable regulatory requirements and with signed agreements
2. The monitor will ensure that all documents relating to the clinical trial procedures, including the protocol, investigator’s brochure, written informed consent, clinical trial reports, and adverse event reporting are accurate, complete, and adhere to the requirements of GCP, the ethics committee (EC), and the applicable regulatory requirements
3. The monitor will also be responsible for:
  - Communication with the principal investigator and verification that the trial site has the resources to conduct the trial and that each Investigator is enrolling only eligible subjects
  - Checking the accuracy of the source documents completed by the investigator(s)

### Adverse event reporting and harms {22}

Any unexpected adverse events will be reported to the relevant authorities as outlined below and will be followed up vigilantly by the investigators and the sponsor.

The sponsor will promptly notify all relevant investigators/institutions and the regulatory authorities of any findings that could adversely affect the safety of subjects, impact the conduct of the trial, or alter the EC’s approval/favorable opinion to continue the trial.

The investigator(s) will monitor each subject for clinical evidence of adverse events (Aes) on a routine basis throughout the study and for up to 36 months. The investigator(s) will assess and record any vascular and non-vascular adverse event as well as related information such as the date of onset, description, final diagnosis/syndrome (if known), severity, time course, duration and outcome, and relationship of the adverse event to the study treatment.

The investigative site is responsible for reporting all serious and/or unexpected events to the applicable EC, monitor, and sponsor by telephone and email within 24 h. The investigative site is responsible for maintaining a copy of the report in the subject’s study file. In addition, the sponsor and the investigative site may be required to report serious and/or unexpected events directly to BfArM [Bundesinstituts für Arzneimittel und Medizinprodukte (Federal Institute for Drugs and Medical Devices)].

Any observed or reported serious adverse events (SAE) which is considered unexpected and related to the study procedures (definitely, probably or possibly) must be reported by the investigative site to the monitor and sponsor within 24 h by telephone. The EC will be notified within 72 h with comment from the principal investigator. The initial written report should include but should not be limited to:

- Subject information, including relevant identifiers
- Study device information, including batch number
- Event onset date and time
- Event description and level of severity
- The investigator’s opinion of causality
- Etiology
- Details on reporter of event

All SAEs will be followed by the investigative site to satisfactory resolution.

### Frequency and plans for auditing trial conduct {23}

The study monitoring plan, recruitment rate, study compliance, and findings from previous visits determine the frequency of regular and interim visits.

### Plans for communicating important protocol amendments to relevant parties (e.g., trial participants, ethical committees) {25}

The protocol, the informed consent document, and relevant supporting information must be submitted to the EC for review and must be approved before the study is initiated. The principal investigator is responsible for keeping the EC informed of the progress of the study and of any changes made to the protocol as deemed appropriate, but in any case, the EC must be updated at least once a year. The principal investigator must also keep the EC informed of any significant Aes. The principal investigator is required to promptly notify the EC of all adverse events that are both serious and unexpected.

### Dissemination plans {31a}

Progress reports and a final report at the end of the study will be prepared under the responsibility of the sponsor and will be submitted to the reviewing ethics committees in accordance with local regulations. Publication in a peer-reviewed journal is also planned.

## Discussion

This prospective, randomized, multicenter trial is designed to evaluate the acute and intermediate outcomes of atherectomy plus DCB (optionally with provisional stenting) compared with open surgery (endarterectomy) in the treatment of de novo atherosclerotic CFA lesions. All devices are commercially available and will be used in accordance with the manufacturer’s instructions. A non-inferiority hypothesis is assumed for the endovascular procedure compared to open surgery.

PTA has been shown to be a successful method of treating PAD and is established as one of the first line therapy options in most arterial regions [1].

Today, the use of stents, directional atherectomy, and DCB leads to favorable mid-term results after endovascular procedures in the femoropopliteal arteries [2, 3, 12]. However, for CFA lesions, open vascular surgery (endarterectomy) remains the treatment of choice. Reported 1-year primary patency rates after CFA endarterectomy are between 85 and 95% [4]. However, open surgery has been associated with up to 5% major adverse events and 20% minor complications, including infection, acute reocclusion, hematoma, and nerve damage [4–6].

In the past, endovascular treatment of CFA was only performed in exceptional cases, e.g., in patients with multilevel disease, in patients who are not suitable for surgery, or in patients who refuse open surgery.

In 2011, Bonvini et al. published results of a prospectively maintained single-center database of 360 consecutive CFA interventions. Balloon angioplasty was performed as the primary intervention in nearly all cases (98.6%), whereas additional stenting was needed in 133 procedures (36.9%). The overall 1-year primary patency rate was 72.4%. Subgroup analyses showed higher patency rates in patients treated with stent implantation (80%) and atherectomy (88.2%). However, the group sizes were too small to draw a definitive conclusion [13].

In the meantime, several studies show that endovascular therapy may have the potential to replace open surgery at least for some anatomical characteristics of CFA lesions [7–9, 14–16].

The TECCO study, a prospective, randomized, multicenter trial comparing primary stenting with open surgery of CFA lesions, showed comparable reintervention rates at 2 years [7].

Bath et al. performed a pooled analysis including 20 studies with a total of 836 patients and 897 CFA interventions. Technical success was 95%. Balloon angioplasty alone was undertaken in 68.8% of cases and stenting in 22.3%. Primary patency at 12 months was 77%. Subgroup analysis revealed a significantly higher mean primary patency for routine stenting compared to a selective stenting strategy (91.4% versus 75%; *P* < 0.05) [14].

In a retrospective, single-center study by Böhme et al., 250 patients with CFA lesions were treated with stent implantation. The primary end point was the target lesion revascularization (TLR) rate. Median follow-up was 21 months (average 19.2 ± 7.8). In total, 41 patients (16.4%) needed a TLR. The primary patency rate was 81.2% at 12 months [15].

In another retrospective analysis by Allan et al., thirty-nine interwoven stents were deployed in 33 patients, and 56 surgical endarterectomies were performed in 55 patients. No significant differences were noted in primary patency (95.5% vs 94.4%, *P* = 0.618), major adverse limb events (5.1% vs 5.4%, *P* = 0.949), and all-cause mortality (14.1% vs 3.6%, *P* = 0.076) between the treatment groups at 12 months [16].

Despite these promising data, there are concerns about stent implantation because of the potential stress on the CFA due to its location in a motion segment potentially resulting in stent deformation or vessel damage. Moreover, a “leave no metal behind” strategy preserves the native CFA as an arterial access for percutaneous interventions and avoids complicated surgical repair of this vascular section (e.g., future endarterectomy or bypass surgery) due to a previously implanted stent.

In this context, the combination of atherectomy and balloon angioplasty is an endovascular option for treating these lesions without or minimal stenting. However, there is limited data on the performance of atherectomy in CFA disease [8, 9]. Cioppa et al. reported a TLR rate of 3.3% after 12 months [8]. In a study with partial DCB application after atherectomy, the TLR rate was 13.6% with a mean follow-up time of 31.0 ± 21.6 months [9].

Whether open surgery is superior to these current endovascular therapy options was not previously investigated in a prospective, randomized trial. Endovascular therapy would be a minimally invasive option for the treatment of atherosclerotic CFA lesions if the PESTO-CFA trial demonstrates non-inferiority of atherectomy combined with DCB angioplasty.

## Trial status

Protocol version number V5.3, 12 May 2023.

Study start: January 2017.

Estimated primary completion: December 2027.

## Data Availability

The coordinating center will have access to the final dataset of the study. Upon reasonable request, the full study protocol can be obtained from the corresponding author.

## Appendix

### Participating sites

**Table:**

Universitätsklinikum Freiburg im Breisgau/UHZ Bad Krozingen
Universität Leipzig
Klinikum Arnsberg GmbH
St. Franziskus-Hospital GmbH Münster
SRH Klinikum Karlsbad-Langensteinbach GmbH
Fürst-Stirum-Ktinik Bruchsal/Städtisches Klinikum Karlsruhe gGmbH
Gefäßzentrum der Elblandkliniken mit den Standorten Radebeul und Riesa
Klinikverbund Kempten-Oberallgäu gGmbH Klinik Immenstadt
Kantonsspital Aarau
Universitätsklinikum Heidelberg
GRN-Klinik Weinheim
Luzerner Kantonspital

### Clinical categories [12]

**Table:**

Grade	Category	Clinical description	Objective criteria
	0	Asymptomatic, no hemodynamically significant occlusive disease	Normal results of treadmill/stress test
I	1	Mild claudication	Treadmill exercise completed, postexercise AP is greater than 50 mmHg but more than 25 mmHg less than normal
	2	Moderate claudication	Symptoms between those of categories 1 and 3
	3	Severe claudication	Treadmill exercise cannot be completed, postexercise AP is less than 50 mmHg
II	4	Ischemic rest pain	Resting AP of 40 mmHg or less, flat or barely pulsatile ankle or metatarsal plethysmographic tracing, toe pressure less than 30 mmHg
III	5	Minor tissue loss, nonhealing ulcer, focal gangrene with diffuse pedal ischemia	Resting AP of 60 mmHg or less, ankle or metatarsal plethysmographic tracing flat or barely pulsatile, toe pressure less than 40 mmHg
	6	Major tissue loss, extending above transmetatarsal level, functional foot no longer salvageable	Same as for category 5

*AP* ankle pressure.

## Abbreviations

ABI: Ankle-brachial index
ASA: Acetylsalicylic acid
CFA: Common femoral artery
CRF: Case report form
CRO: Contract research organization
DCB: Drug-coated balloon
DFA: Deep femoral artery
DSMB: Data safety monitoring board
EC: Ethics committee
GCP: Good clinical practice
IFU: Instruction for use
ITT: Intention-to-treat
PAD: Peripheral artery disease
PSV: Peak systolic velocity
PTA: Percutaneous transluminal angioplasty
PVR: Peak velocity ratio
SAE: Serious adverse events
SFA: Superficial femoral artery
TLR: Target lesion revascularization
WIQ: Walking Impairment Questionnaire
